# Circ_0116061 regulated the proliferation, apoptosis, and inflammation of osteoarthritis chondrocytes through regulating the miR-200b-3p/SMURF2 axis

**DOI:** 10.1186/s13018-021-02391-9

**Published:** 2021-04-13

**Authors:** Wei Zheng, Guanhua Hou, Yong Li

**Affiliations:** 1 Department of Joint Surgery, Rizhao Central Hospital, Rizhao, 276800 Shandong China; 2 Department of Orthopedics, Peking University Medical Zibo Hospital, Zibo, 255069 Shandong China; 3 Department of Spine, Central People’s Hospital of Tengzhou, 181 Xingtan Road, Tengzhou, 277500 Shandong China

**Keywords:** Osteoarthritis, Chondrocytes, Circ_0116061, MiR-200b-3p, SMURF2

## Abstract

**Background:**

Circular RNA (circRNA) has been shown to be associated with osteoarthritis (OA) progression. Circ_0116061 has been found to be highly expressed in OA cartilage tissues, but its role and mechanism in OA progression remain unclear.

**Methods:**

Expression levels of circ_0116061, microRNA (miR)-200b-5p, and Smad ubiquitin regulatory factor 2 (SMURF2) were detected using quantitative real-time PCR. The proliferation and apoptosis of cells were measured using cell counting kit 8 (CCK8) assay, colony formation assay, and flow cytometry. Furthermore, the protein levels of proliferation-related marker, apoptosis-related markers, inflammatory factors, and SMURF2 were tested using western blot (WB) analysis. In addition, the interaction between miR-200b-3p and circ_0116061 or SMURF2 was examined using dual-luciferase reporter assay and biotin-labeled RNA pull-down assay.

**Results:**

Circ_0116061 and SMURF2 were highly expressed, and miR-200b-3p was lowly expressed in OA cartilage tissues. Knockdown of circ_0116061 could promote the proliferation and inhibit the apoptosis and inflammation of OA chondrocytes. MiR-200b-3p could be sponged by circ_0116061, and its inhibitor could reverse the regulation of circ_0116061 silencing on the biological functions of OA chondrocytes. SMURF2 was a target of miR-200b-3p, and its expression was positively regulated by circ_0116061. Silencing of SMURF2 also could enhance the proliferation and suppress the apoptosis and inflammation of OA chondrocytes. Furthermore, the regulation of circ_0116061 silencing on the biological functions of OA chondrocytes also could be reversed by SMURF2 overexpression.

**Conclusion:**

Our data showed that circ_0116061 might regulate the miR-200b-3p/SMURF2 axis to promote the progression of OA.

## Highlights

1. Circ_0116061 silencing enhances the proliferation and represses apoptosis and inflammation of OA chondrocytes.
2. Circ_0116061 sponges miR-200b-3p.
3. MiR-200b-3p targets SMURF2.

## Introduction

Osteoarthritis (OA) is a chronic degenerative joint disease caused by articular cartilage degradation, subchondral bone sclerosis, osteophyte formation, synovial inflammation, meniscal degeneration, and inflammation and fibrosis of the infrapatellar fat pad [1–5]. OA has become a major public health problem, and the loss of joint function caused by it seriously affects the quality of life of the elderly [6, 7]. The onset of OA is closely related to the apoptosis of chondrocytes and the persistent pathological inflammation in the joints [8, 9]. Therefore, finding effective molecular targets for regulating chondrocyte apoptosis and inflammation is expected to offer new ideas for alleviating OA progression.

In recent years, the important role of non-coding RNA, including circular RNA (circRNA) and microRNA (miRNA), in a variety of diseases has been widely confirmed. Importantly, the function of circRNA as a miRNA sponge also provides a way to elucidate circRNA mechanism [10, 11]. Many circRNAs have been identified as potential biomarkers for the diagnosis and treatment of OA, such as circRNA.33186 [12], circSERPINE2 [13], and circRNA-UBE2G1 [14]. Zhao et al. used high-throughput sequencing to find that circ_0136474 was highly expressed in OA cartilage tissues, and confirmed that it could promote OA progression by inhibiting the proliferation and promoting apoptosis and inflammation of OA chondrocytes [15]. In their study, they also screened that circ_0116061 was also an upregulated circRNA in OA cartilage tissues [15]. However, whether circ_0116061 is involved in the regulation of OA progression has not been studied.

MiR-200b-3p has been confirmed to participate in the regulation of cancer malignant progression as a tumor suppressor, including melanoma [16] and hepatocellular carcinoma [17]. It had been reported that miR-200b-3p was significantly low expressed in OA cartilage tissues and chondrocytes, which overexpression could increase OA chondrocyte viability and suppress apoptosis [18]. Therefore, miR-200b-3p might be the key miRNA that regulated the development of OA. Smad ubiquitin regulatory factor 2 (SMURF2) is a gene that is highly expressed in OA cartilage tissue and chondrocytes and has been shown to be closely related to OA progression [19, 20].

The purpose of this study is to explore the role of circ_0116061 in the progression of OA and to reveal its underlying molecular mechanism through the hypothesis of circRNA/miRNA/mRNA axis. Our data showed circ_0116061 might play an active role in OA progression. Additionally, we found that there had been correlations among circ_0116061, miR-200b-3p, and SMURF2 expression in OA cartilage tissues. Further analysis revealed that circ_0116061 could regulate SMURF2 by sponging miR-200b-3p. Therefore, our research putted forward the hypothesis that circ_0116061 regulated OA progression via miR-200b-3p/SMURF2.

## Materials and methods

### Cartilage tissues

Our research was approved by Rizhao Central Hospital. Knee cartilage tissues were collected from OA patients (*n* = 37) and healthy controls (*n* = 19, only fractures) in Rizhao Central Hospital. For this study, all patients signed an informed consent. The clinical parameters of healthy controls and OA patients are shown in Table 1.

**Table 1:** Clinical parameters of healthy controls and OA patients

Clinical parameter	Healthy controls (*n* = 19)	OA patients (*n* = 37)	KL grade II (*n* = 9)	KL grade III (*n* = 16)	KL grade IV (*n* = 12)
Age (years)	59 ± 6	63 ± 9	64 ± 7	65 ± 7	64 ± 8
BMI (kg/m^2^)	25.1 ± 2.6	25.5 ± 2.9	25.3 ± 3.6	25.7 ± 2.7	25.9 ± 2.1
Gender (F/M)	12/7	23/14	6/3	9/7	8/4
ESR, mean (mm/h)	14.1 ± 3.7	27.1 ± 5.2	25.3 ± 4.9	28.1 ± 4.3	28.5 ± 3.9
CRP, mean (mg/L)	5.2 ± 2.6	13.2 ± 4.9	12.8 ± 5.6	13.6 ± 4.5	13.9 ± 3.9

*OA* osteoarthritis, *KL* Kellgren-Lawrence, *BMI* body mass index, *ESR* erythrocyte sedimentation rate, *CRP* C-reactive protein

### Cell culture

OA chondrocytes were separated from OA cartilage tissues according to the previous study [15]. OA chondrocytes were grown in DMEM medium (Gibco, Carlsbad, CA, USA) containing 10% fetal bovine serum (FBS; Gibco) and 1% penicillin-streptomycin liquid (Gibco) at 37 °C in a 5% CO_2_ incubator.

### Cell transfection

Cell transfections were all performed with Lipofectamine 3000 (Invitrogen, Carlsbad, CA, USA) following the manufacturer’s protocols. The small interfering RNA against circ_0116061 and SMURF2 (si-circ_0116061 and si-SMURF2) or negative control (si-NC), miR-200b-3p mimic and inhibitor (miR-200b-3p and anti-miR-200b-3p) or their negative controls (miR-NC and anti-NC), and SMURF2 overexpression vector (OE-SMURF2) and negative control (OE-NC) were obtained from Ribobio (Guangzhou, China). The concentrations of oligonucleotides were 50 nM and those of vectors were 2 μg/mL.

### Quantitative real-time PCR (qRT-PCR)

Total RNA was isolated with RNeasy Mini Kit (Qiagen, Duesseldorf, Germany), and cDNA was then synthesized using RevertAid Reverse Transcriptase (Sangon, Shanghai, China). Real-time PCR was performed in a PCR system using Universal SYBR Green Master (Roche, Basel, Switzerland). Relative expression were normalized to GAPDH (for circ_0116061 and SMURF2) or U6 (for miR-200b-3p) and calculated by the 2^−ΔΔCt^ method. The primers were shown as below: circ_0116061, F 5′-AGCACGGATTTGGAGATTTG-3′, R 5′-GGCAGATTTGCAAAAGATGA-3′; miR-200b-3p, F 5′-GCCGAGTAATACTGCCTGGTAA-3′, R 5′-CTCAACTGGTGTCGTGGAG-3′; SMURF2, F 5′-GGCAATGCCATTCTACAGATACT-3′, R 5′-CAACCGAGAAATCCAGCACCT-3′; GAPDH, F 5′-GGAAGGTGAAGGTCGGAGTC-3′, R 5′-CGTTCTCAGCCTTGACGGT-3′; U6, F 5′-CTCGCTTCGGCAGCACATATACT-3′, R 5′-ACGCTTCACGAATTTGCGTGTC-3′.

### Cell counting kit 8 (CCK8) assay

CCK8 assay was performed to measure cell viability. After transfecting with oligonucleotides and vectors, OA chondrocytes were reseeded into 96-well plates (2 × 10^6^ cells/well) and cultured for 24, 48, and 72 h. Then, the CCK8 solution (Dojindo, Kumamoto, Japan) was incubated with OA chondrocytes for 4 h. The optical density (OD) value at 450 nm was recorded using a microplate reader (Biotek, Winooski, Vermont, USA) to assess cell viability.

### Colony formation assay

OA chondrocytes (200 cells) were seeded in the cell culture dish and cultured for 14 days. The cloned cells were fixed with ethanol (KeyGen, Jiangsu, China) and stained with crystal violet (KeyGen). Cell cloning number was counted under a microscope (Olympus, Tokyo, Japan).

### Flow cytometry

Cell apoptosis was determined using Annexin V-FITC Apoptosis Detection Kit (Dojindo). In brief, OA chondrocytes were collected and suspended with binding buffer at a concentration of 1 × 10^6^ cells/mL. Then, cell suspensions were stained with Annexin V-FITC and propidium iodide. The apoptosis was analyzed by a flow cytometer (BD Biosciences, San Jose, CA, USA).

### Western blot (WB) analysis

OA chondrocytes were treated with RIPA lysis buffer (Applygen, Beijing, China). After quantifying the protein concentration, 30 μg protein was separated by SDS-PAGE gel and transferred to PVDF membranes (Beyotime, Shanghai, China). The membranes were blocked with nonfat milk and then probed with primary antibodies against Cyclin D1 (1:10,000), Bax (1:10,000), Cleaved-caspase-3 (Cleaved-casp3, 1:500), Bcl2 (1:2000), interleukin (IL)-1β (1:1000), IL-6 (1:2000), IL-1α (1:6000), tumor necrosis factor α (TNFα, 1:2000), SMURF2 (1:1000), or GAPDH (1:2500). After incubating at 4 °C overnight, the membranes were incubated with goat anti-rabbit IgG (1:50,000) for 2 h. Finally, an ECL luminescence reagent (Sangon) was used for protein detection. All antibodies were from Abcam (Cambridge, MA, USA).

### Dual-luciferase reporter assay

Based on the binding sites of miR-200b-3p in circ_0116061 and SMURF2 3′UTR, the wild-type (wt) and mutant-type (mut) sequences of circ_0116061 and SMURF2 3′UTR were inserted into the psi-CHECK-2 vector (Promega, Madison, WI, USA), respectively. HEK293 cells (Procell, Wuhan, China) were inoculated into a 24-well plate (5 × 10^4^ cells/well). After the cells were grown to 60% confluences, the reporter vectors were transfected into the cells with miR-200b-3p mimic or miR-NC for 48 h. Using the Dual-Luciferase Reporter Assay System Kit (Promega), the relative luciferase activity was detected.

### Biotin-labeled RNA pull-down assay

The bio-miR-200b-3p probe and control probe (bio-miR-NC) were synthesized by Sangon. After transfecting with bio-miR-200b-3p or bio-miR-NC, OA chondrocytes were harvested and lysed. The cell lysates were incubated with Dynabeads M-280 Streptavidin (Invitrogen), and then the purified RNA was conducted to measure circ_0116061 expression.

### Statistical analysis

GraphPad Prism 6.0 software (GraphPad Inc., La Jolla, CA, USA) was used to conduct the statistical analysis. All data were presented as mean ± standard deviation from 3 independent experiments. Results from different groups were analyzed by one-way analysis of variance or Student’s *t*-test. Pearson correlation analysis was used for analyzing the correlations among circ_0116061, miR-200b-3p, and SMURF2. *P* < 0.05 was considered statistically significant.

## Results

### The expression of circ_0116061, miR-200b-3p, and SMURF2 in OA cartilage tissues

**Fig. 1:**
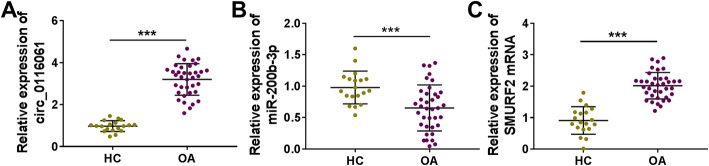
The expression of circ_0116061, miR-200b-3p, and SMURF2 in cartilage tissues. The expression levels of circ_0116061 (**a**), miR-200b-3p (**b**), and SMURF2 (**c**) in the cartilage tissues of healthy controls and OA patients were detected by qRT-PCR. ***P < 0.001

Using qRT-PCR, we measured the expression of circ_0116061, miR-200b-3p, and SMURF2 in the cartilage tissues of OA patients and healthy controls. Compared to the cartilage tissues of healthy controls, we found that circ_0116061 was notably enhanced (Fig. 1a), miR-200b-3p was significantly decreased (Fig. 1b), and SMURF2 was markedly promoted (Fig. 1c) in the cartilage tissues of OA patients. Therefore, we speculated that circ_0116061, miR-200b-3p, and SMURF2 might play key roles in the development of OA.

### Circ_0116061 silencing promoted the proliferation and inhibited the apoptosis and inflammation of OA chondrocytes

**Fig. 2:**
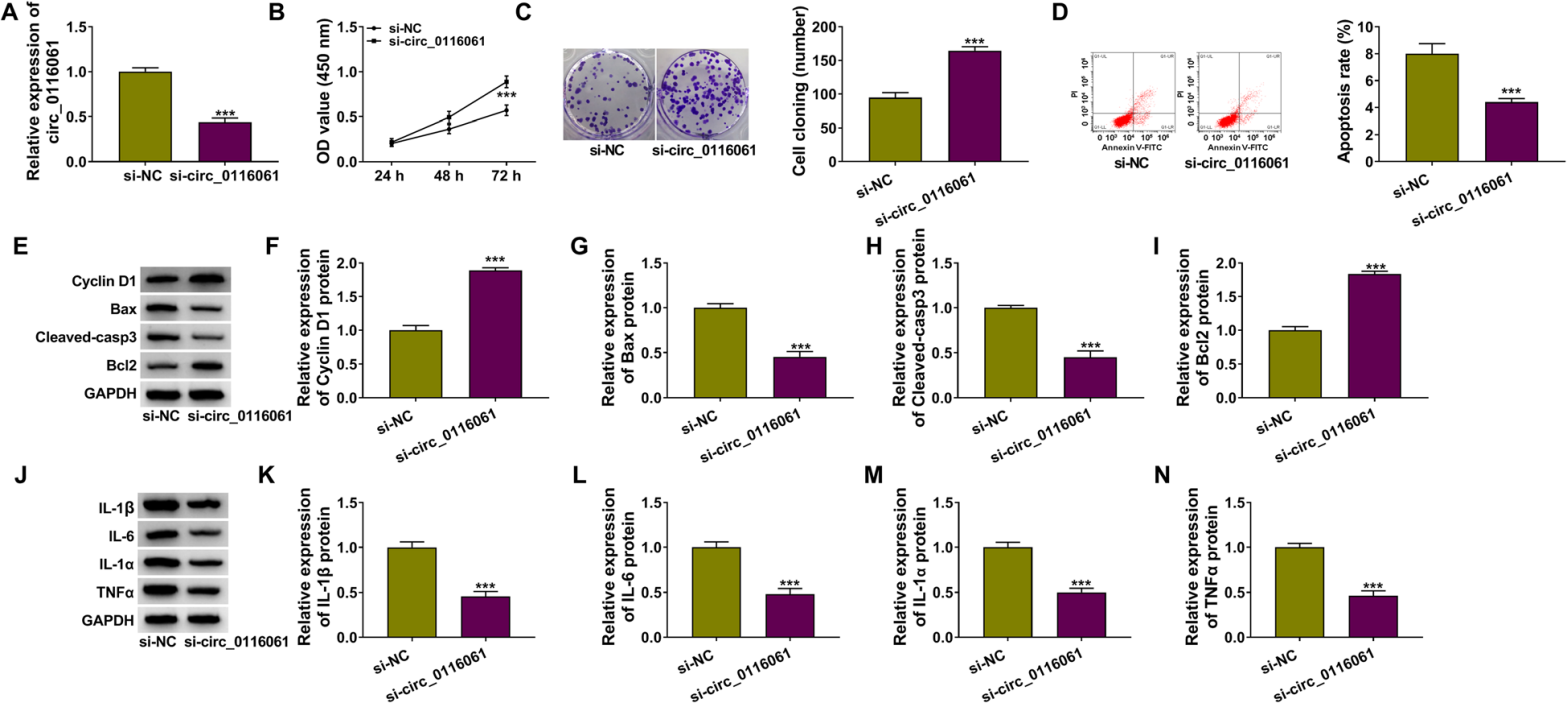
Silencing of circ_0116061 regulated the biological functions of OA chondrocytes. OA chondrocytes were transfected with si-NC or si-circ_0116061. **a** The expression of circ_0116061 was detected by qRT-PCR. CCK8 assay (**b**), colony formation assay (**c**), and flow cytometry (**d**) were used to test cell viability, the cell cloning number, and apoptosis rate. **e**–**n** The protein levels of Cyclin D1, Bcl2, Bax, Cleaved-casp3, IL-1β, IL-6, IL-1α, and TNFα were determined by WB analysis. ***P < 0.001

To explore the role of circ_0116061 in OA, we silenced circ_0116061 expression using si-circ_0116061. The decreased expression of circ_0116061 indicated the transfection efficiency of si-circ_0116061 in OA chondrocytes was good (Fig. 2a). CCK8 results suggested that circ_0116061 knockdown could increase the viability of OA chondrocytes (Fig. 2b). Moreover, silenced circ_0116061 also markedly promote the cell cloning number in OA chondrocytes (Fig. 2c). The results of flow cytometry showed that the apoptosis rate of OA chondrocytes was obviously repressed in the presence of circ_0116061 silencing (Fig. 2d). In addition, we also measured the protein levels of Cyclin D1, Bax, Cleaved-casp3, and Bcl2 and found that circ_0116061 knockdown remarkably increased the protein levels of Cyclin D1 and Bcl2, while decreasing the protein levels of Bax and Cleaved-casp3 in OA chondrocytes (Fig. 2e–i). Furthermore, the protein levels of inflammatory factors (IL-1β, IL-6, IL-1α, and TNFα) were obviously reduced in OA chondrocytes downregulating circ_0116061 (Fig. 2j–n). These data confirmed that circ_0116061 knockdown could alleviate OA progression.

### Circ_0116061 could serve as a sponge for miR-200b-3p

**Fig. 3:**
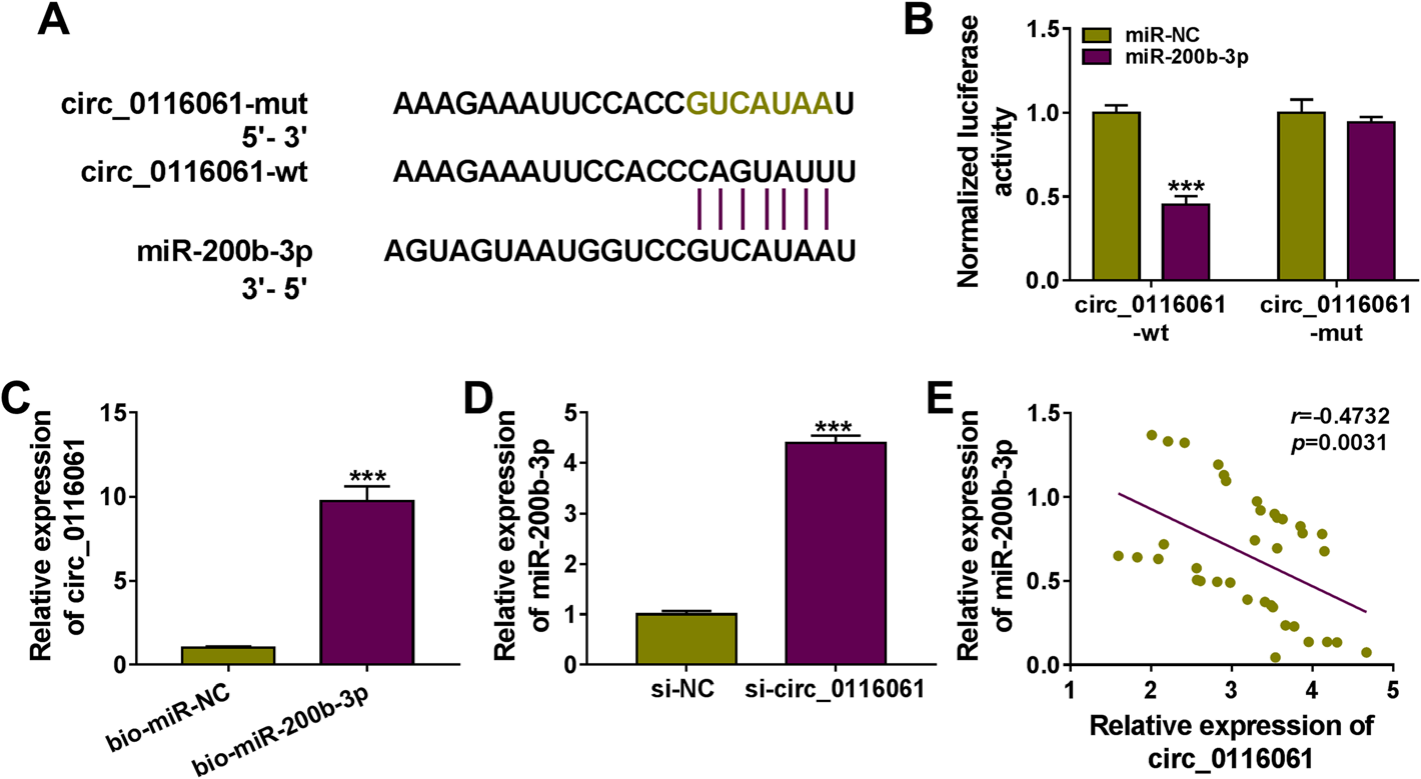
Circ_0116061 sponged miR-200b-3p. **a** The binding sites between miR-200b-3p and circ_0116061 were shown. Dual-luciferase reporter assay (**b**) and biotin-labeled RNA pull-down assay (**c**) were performed to measure the interaction between miR-200b-3p and circ_0116061. **d** After transfecting with si-NC or si-circ_0116061 into OA chondrocytes, the expression of miR-200b-3p was detected by qRT-PCR. **e** Pearson correlation analysis was used to analyze the correlation between miR-200b-3p and circ_0116061 in OA cartilage tissues. ***P < 0.001

Using the bioinformatics software analysis (Circinteractome: https://circinteractome.nia.nih.gov/), we discovered that miR-200b-3p had complementary binding sites with circ_0116061 (Fig. 3a). The results of the dual-luciferase reporter assay indicated that miR-200b-3p overexpression could repress the luciferase activity of circ_0116061-wt reporter vector, while there was no effect on the luciferase activity of circ_0116061-mut reporter vector (Fig. 3b). Biotin-labeled RNA pull-down assay suggested that the expression of circ_0116061 was notably enriched in the bio-miR-200b-3p probe compared to the bio-miR-NC probe (Fig. 3c). Meanwhile, we also discovered that circ_0116061 silencing could enhance the expression of miR-200b-3p in OA chondrocytes (Fig. 3d), and there was a negative correlation between circ_0116061 and miR-200b-3p in OA cartilage tissues (Fig. 3e).

### MiR-200b-3p inhibitor reversed the regulation of circ_0116061 silencing on the proliferation, apoptosis, and inflammation of OA chondrocytes

**Fig. 4:**
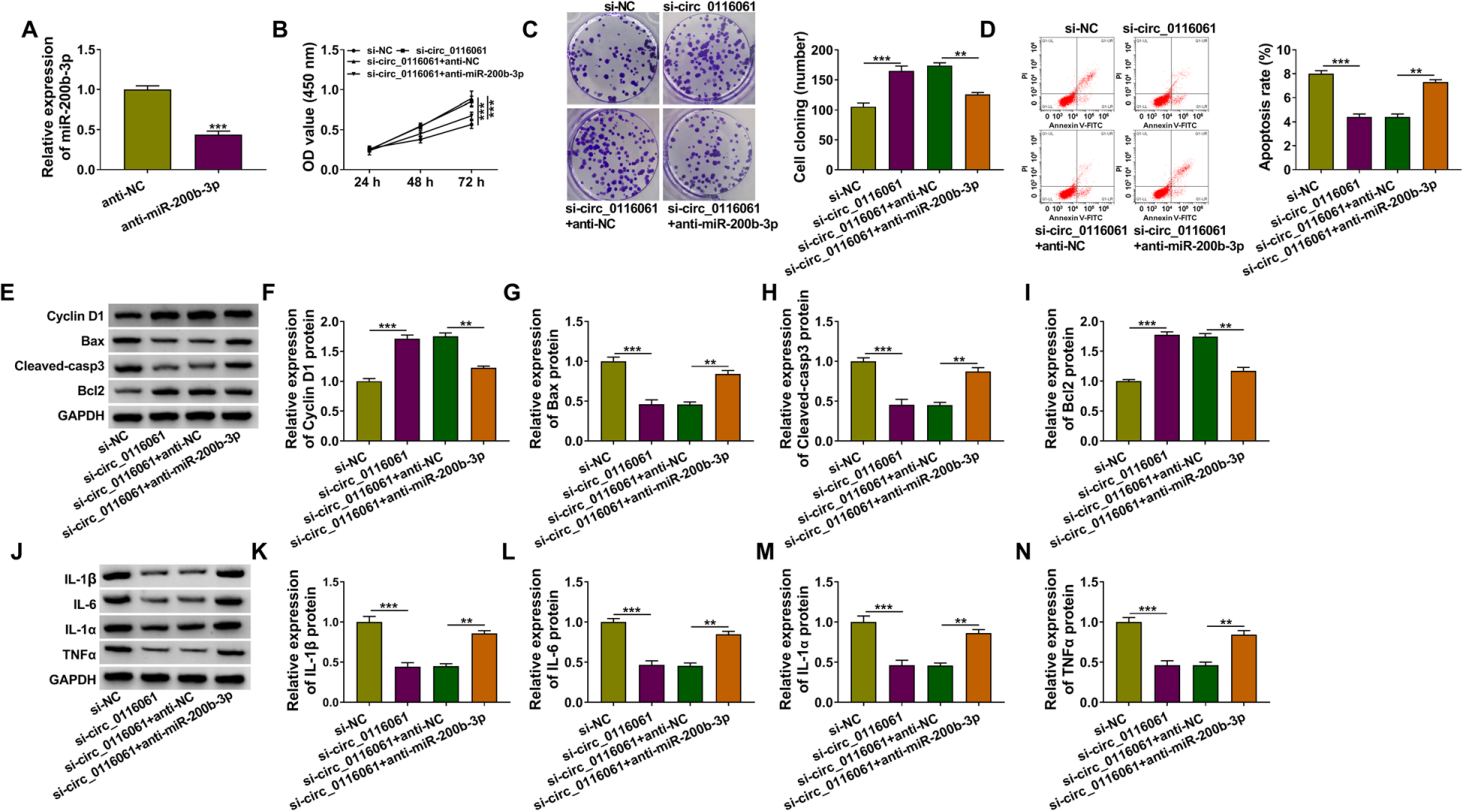
Effects of circ_0116061 silencing and miR-477 inhibitor on the biological functions of OA chondrocytes. **a** The transfection efficiency of anti-miR-200b-3p was evaluated by detecting miR-200b-3p expression using qRT-PCR. **b**–**n** OA chondrocytes were transfected with si-NC, si-circ_0116061, si-circ_0116061 + anti-NC or si-circ_0116061 + anti-miR-200b-3p. Cell viability, the cell cloning number, and apoptosis rate were determined using CCK8 assay (**b**), colony formation assay (**c**), and flow cytometry (**d**). **e**–**n** WB analysis was used to examine the protein levels of Cyclin D1, Bcl2, Bax, Cleaved-casp3, IL-1β, IL-6, IL-1α, and TNFα. **P < 0.01, ***P < 0.001

Subsequently, we constructed the miR-200b-3p inhibitor and confirmed that anti-miR-200b-3p could inhibit miR-200b-3p expression in OA chondrocytes (Fig. 4a). Then, si-circ_0116061 and anti-miR-200b-3p were co-transfected into OA chondrocytes to confirm whether circ_0116061 regulated OA progression by sponging miR-200b-3p. Using CCK8 and colony formation assay, we found that miR-200b-3p inhibitor could reverse the promotion effect of circ_0116061 silencing on the viability and the cell cloning number in OA chondrocytes (Fig. 4b, c). Moreover, the suppressive effect of circ_0116061 knockdown on the apoptosis rate of OA chondrocytes also could be abolished by a miR-200b-3p inhibitor (Fig. 4d). Additionally, the increasing effect of circ_0116061 silencing on the protein levels of Cyclin D1 and Bcl2, as well as the decreasing effect on the protein levels of Bax, Cleaved-casp3, IL-1β, IL-6, IL-1α, and TNFα, also could be reversed by the miR-200b-3p inhibitor (Fig. 4e–n). All data indicated that circ_0116061 might sponge miR-200b-3p to regulate OA progression.

### Circ_0116061 sponged miR-200b-3p to target SMURF2

**Fig. 5:**
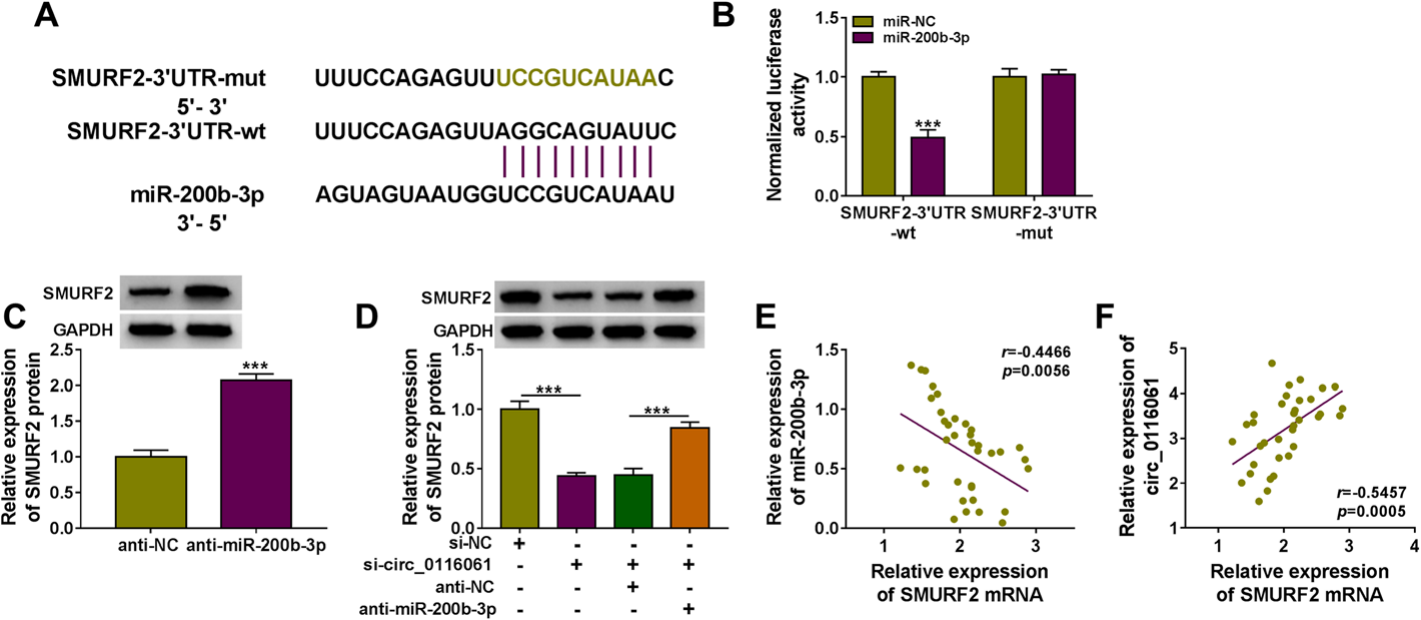
miR-200b-3p targeted SMURF2. **a** The binding sites between SMURF2 3′UTR and miR-200b-3p were presented. **b** The interaction between SMURF2 and miR-200b-3p was confirmed by a dual-luciferase reporter assay. **c** After transfecting with anti-NC or anti-miR-200b-3p into OA chondrocytes, the protein expression of SMURF2 was examined using WB analysis. **d** OA chondrocytes were transfected with si-NC, si-circ_0116061, si-circ_0116061 + anti-NC, or si-circ_0116061 + anti-miR-200b-3p. The protein expression of SMURF2 was determined by WB analysis. **e**–**f** The correlation between SMURF2 and miR-200b-3p or circ_0116061 was determined using Pearson correlation analysis. ***P < 0.001

Surprisingly, the binding sites between SMURF2 3′UTR and miR-200b-3p were predicted using the Targetscan software (http://www.targetscan.org/vert_72/) (Fig. 5a). MiR-200b-3p overexpression also could inhibit the luciferase activity of SMURF2-3′UTR-wt vector without affecting that of the SMURF2-3′UTR-mut vector (Fig. 5b). After inhibiting the expression of miR-200b-3p using anti-miR-200b-3p, we found that the protein level of SMURF2 could be effectively promoted by miR-200b-3p downregulation (Fig. 5c). Also, circ_0116061 knockdown had an inhibitory effect on SMURF2 expression, while this effect could be reversed by miR-200b-3p inhibitor (Fig. 5d). In addition, the SMURF2 mRNA expression was negatively correlated with miR-200b-3p and positively correlated with circ_0116061 in OA cartilage tissues (Fig. 5e, f). Our data illuminated that circ_0116061 regulated SMURF2 by sponging miR-200b-3p.

### Knockdown of SMURF2 increased the proliferation, while suppressing the apoptosis and inflammation of OA chondrocytes

**Fig. 6:**
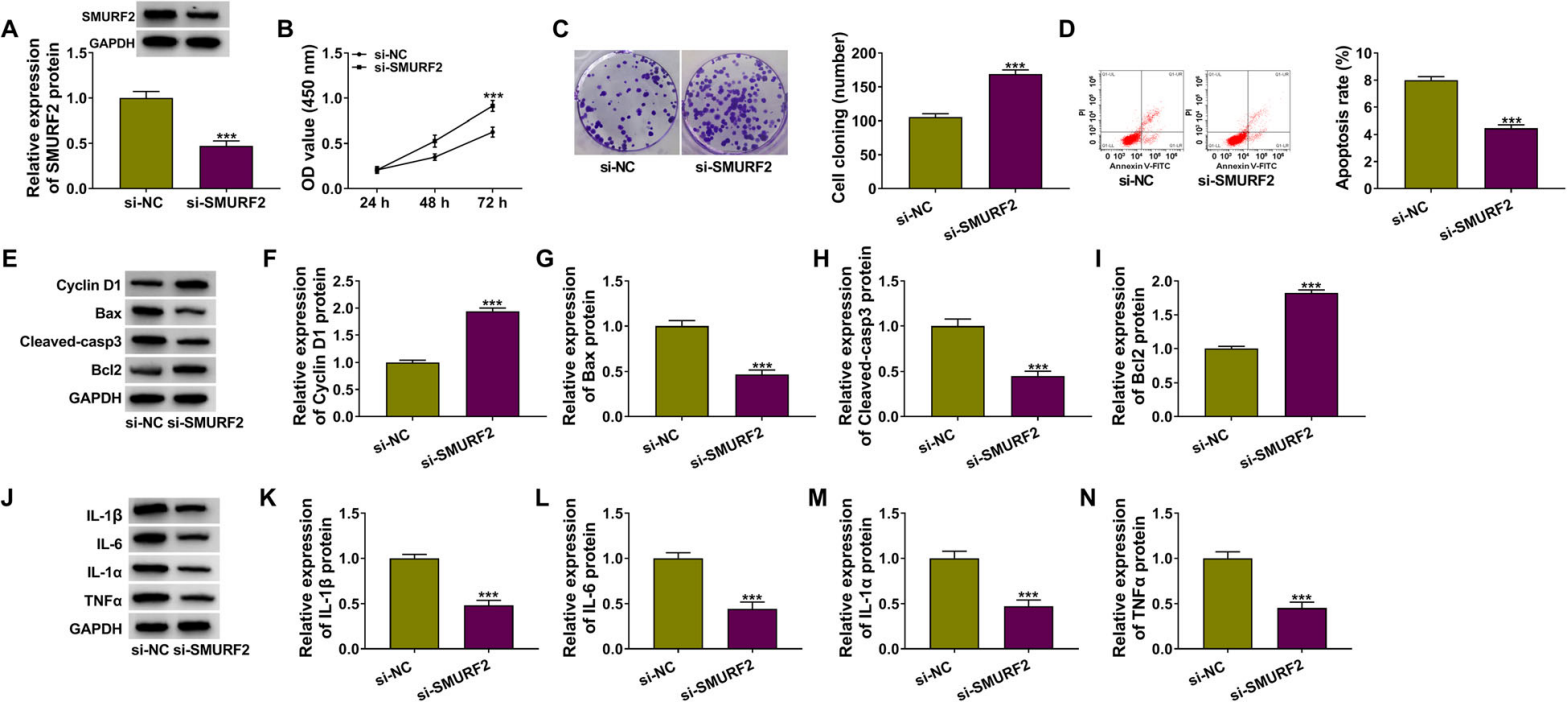
SMURF2 knockdown regulated the biological functions of OA chondrocytes. OA chondrocytes were transfected with si-NC or si-SMURF2. **a** WB analysis was performed to test the protein expression of SMURF2. CCK8 assay (**b**), colony formation assay (**c**), and flow cytometry (**d**) were used to determine the cell viability, the cell cloning number, and apoptosis rate. **e**–**n** WB analysis was employed to test the protein levels of Cyclin D1, Bcl2, Bax, Cleaved-casp3, IL-1β, IL-6, IL-1α, and TNFα. ***P < 0.001

In addition, we also determined the regulation of SMURF2 on the biological functions of OA chondrocytes. After transfecting with si-SMURF2 into OA chondrocytes, we found that the protein level of SMURF2 was remarkably reduced (Fig. 6a). Through measuring OA chondrocyte viability, cell cloning numbers, and apoptosis rate, we confirmed that silenced SMURF2 could promote the proliferation and restrain the apoptosis of OA chondrocytes (Fig. 6b–d). Besides, knockdown of SMURF2 also enhanced the protein levels of Cyclin D1 and Bcl2, while reducing the protein levels of Bax and Cleaved-casp3 in OA chondrocytes (Fig. 6e–i). Furthermore, the protein levels of IL-1β, IL-6, IL-1α, and TNFα in OA chondrocytes also were decreased by silencing SMURF2 (Fig. 6j–n). These results revealed that the role of SMURF2 in OA progression was similar to circ_0116061.

### Overexpressed SMURF2 reversed the regulation of circ_0116061 silencing on OA progression

**Fig. 7:**
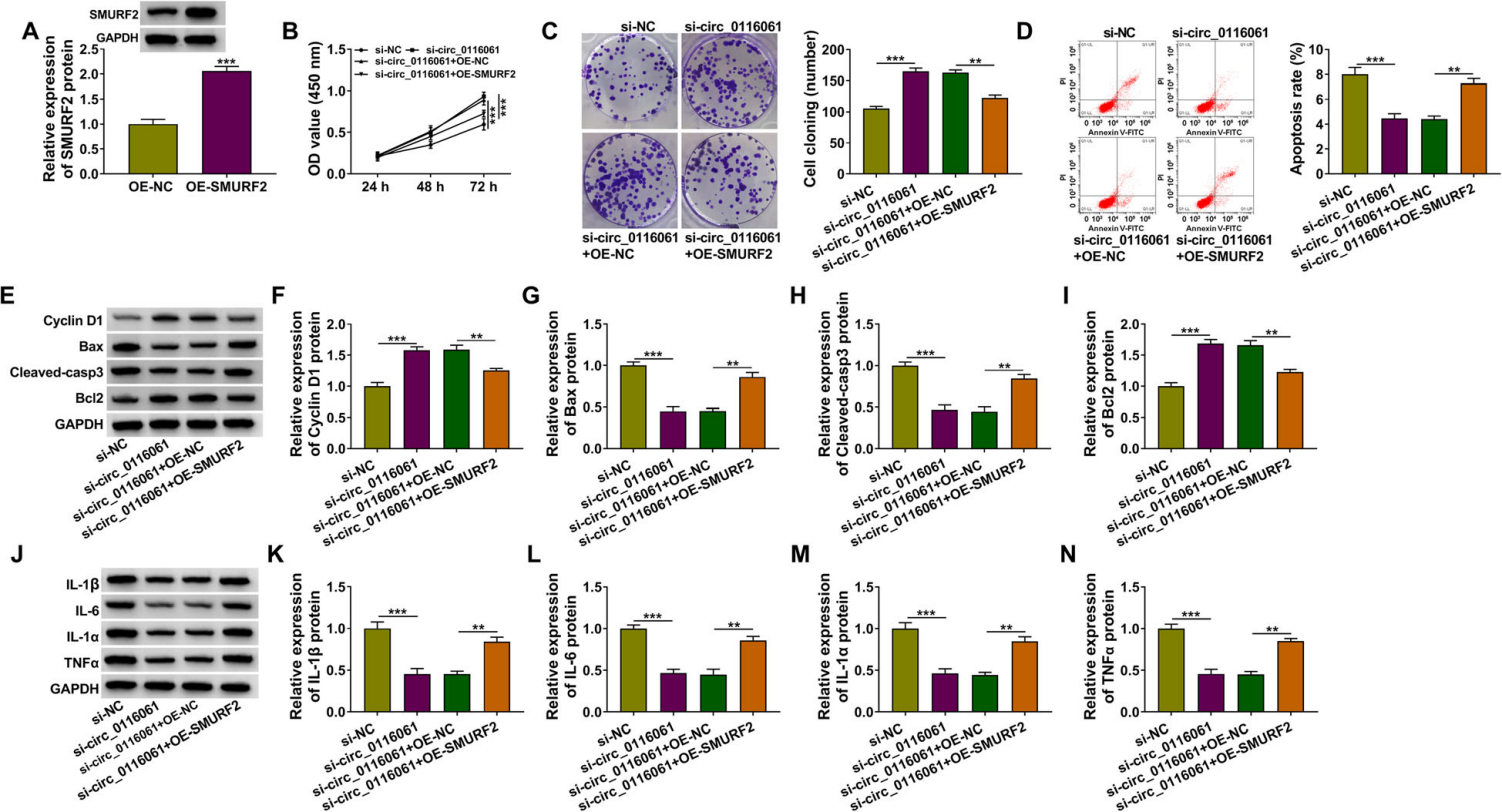
Effects of circ_0116061 silencing and SMURF2 overexpression on the biological functions of OA chondrocytes. **a** OA chondrocytes were transfected with OE-NC or OE-SMURF2. WB analysis was used to measure the protein expression of SMURF2. **b**–**n** OA chondrocytes were transfected with si-NC, si-circ_0116061, si-circ_0116061 + OE-NC, or si-circ_0116061 + OE-SMURF2. Cell viability, the cell cloning number, and apoptosis rate were measured by CCK8 assay (**b**), colony formation assay (**c**), and flow cytometry (**d**). **e**–**n** The protein levels of Cyclin D1, Bcl2, Bax, Cleaved-casp3, IL-1β, IL-6, IL-1α, and TNFα were assessed using WB analysis. **P < 0.01, ***P < 0.001

To further evaluate whether circ_0116061 regulated OA progression via regulating SMURF2 expression, we performed the rescue experiments. The transfection of OE-SMURF2 in OA chondrocytes could significantly promote the expression of SMURF2, which confirmed the good transfection efficiency of OE-SMURF2 (Fig. 7a). Then, si-circ_0116061 and OE-SMURF2 were co-transfected into OA chondrocytes. Function experiments revealed that the addition of OE-SMURF2 could effectively reverse the promotion effect of circ_0116061 knockdown on the viability and the cloning number (Fig. 7b, c), as well as the inhibition effect on the apoptosis rate of OA chondrocytes (Fig. 7d). In addition, the protein levels of Cyclin D1 and Bcl2 enhanced by circ_0116061 silencing, and the protein levels of Bax, Cleaved-casp3, IL-1β, IL-6, IL-1α, and TNFα repressed by circ_0116061 knockdown also could be reversed by the overexpression of SMURF2 (Fig. 7e–n). These results showed that circ_0116061 mainly regulated SMURF2 to mediate the progression of OA.

## Discussion

The occurrence of OA is a complex process involving multiple factors, and its etiology and pathogenesis are still not very clear. A great amount of evidence shows that the degeneration of articular cartilage is considered to be the most important pathological link that causes OA [21, 22]. Chondrocytes, as the only cellular component of cartilage, and their biological characteristics are closely related to the development of OA [23, 24]. Here, we explored the role of a new circRNA, circ_0116061, in the progression of OA by assessing its function in the biological function of OA chondrocytes. Consistent with the previous study [15], our data found that circ_0116061 was overexpressed in OA cartilage tissues and chondrocytes. Interference of circ_0116061 could increase the proliferation and reduce the apoptosis and inflammation of OA chondrocytes, indicating that circ_0116061 silencing might be an effective strategy to alleviate OA progression.

The competitive endogenous RNA (ceRNA) hypothesis provides a new mechanism for the interaction between RNAs. A large number of studies have shown that circRNA can be used as a ceRNA of miRNA to indirectly regulate mRNA expression [25, 26]. For example, circPSMC3 had anti-proliferation and anti-metastasis functions in gastric cancer, which were mainly realized by sponging miR-296-5p [27]. Circ-GRB10 could act as a ceRNA for miR-328-5p to suppress nucleus pulposus cell apoptosis [28]. In addition to being a tumor suppressor, miR-200b-3p had also been found to be related to the progression of endometriosis [29], and its downregulation could also inhibit vascular smooth muscle cell calcification [30]. Here, we discovered that miR-200b-3p was underexpressed in OA cartilage tissues and chondrocytes, which was similar with the past research [18]. MiR-200b-3p inhibitor could reverse the pro-proliferation, anti-apoptosis, and anti-inflammatory functions of circ_0116061 knockdown on OA chondrocytes, confirming that circ_0116061 indeed mediated OA progression via targeting miR-200b-3p.

Additionally, our data showed that SMURF2 was a target of miR-200b-3p and circ_0116061 sponged miR-200b-3p to regulate SMURF2. SMURF2 is a kind of E3 ubiquitin ligase, which can participate in the regulation of TGF-β signaling pathway through the ubiquitin-ribosome pathway [31]. Current research confirmed that SMURF2 was related to the progression of multiple fibrotic diseases, including liver fibrosis [32] and kidney fibrosis [33]. Chen et al. showed that SMURF2 could suppress OA chondrocyte proliferation, while accelerating apoptosis and ECM degradation [19]. Studies had suggested that the high expression of SMURF2 could induce the degeneration of articular chondrocytes and produce the OA-like phenotype [34, 35]. Here, we silenced SMURF2 expression and confirmed that downregulated SMURF2 could facilitate OA chondrocyte proliferation and restrain apoptosis and inflammation. Besides, the reversal effect of SMURF2 on circ_0116061 silencing illuminated that circ_0116061 might positively regulate SMURF2 to mediate OA progression.

Combined with all the results, we proposed that circ_0116061 promoted OA progression through the miR-200b-3p/SMURF2 axis. The pro-proliferation, anti-apoptosis, and anti-inflammatory functions of circ_0116061 silencing on OA chondrocytes suggested that circ_0116061 knockdown might be a beneficial method to alleviate OA progression, which had an important clinical significance.

## Data Availability

All data generated or analyzed during this study are included in this published article.
